# Prevalence and predictors of sleep problems in women following a cancer diagnosis: results from the women’s wellness after cancer program

**DOI:** 10.1007/s11764-023-01346-9

**Published:** 2023-02-24

**Authors:** Shannon L. Edmed, M. Mamun Huda, Simon S. Smith, Charrlotte Seib, Janine Porter-Steele, Debra Anderson, Alexandra L. McCarthy

**Affiliations:** 1 Institute for Social Science Research, The University of Queensland, 80 Meiers Rd, Indooroopilly QLD, Brisbane, 4068 Australia; 2 ARC Centre of Excellence for Children and Families Over the Life Course, The University of Queensland, Brisbane, Australia; 3 ARC Centre of Excellence for the Digital Child, The University of Queensland, Brisbane, Australia; 4 School of Nursing and Midwifery, Griffith University, Southport Queensland, 4215 Australia; 5 School of Nursing, Midwifery and Social Work, The University of Queensland, Brisbane, Australia; 6 The Wesley Hospital Choices Cancer Support Centre, Brisbane, Australia; 7 Faculty of Health, University of Technology Sydney, Sydney, NSW Australia; 8 Mater Research Institute, Brisbane, QLD Australia

**Keywords:** Sleep, Sleep disturbance, Cancer, Women’s cancer, Women’s health

## Abstract

**Purpose:**

Using a discrete dataset from the Women’s Wellness after Cancer Program (WWACP), we examine the prevalence and predictors of self-reported sleep problems in women previously treated for cancer.

**Methods:**

Participants were 351 women (*M*_age_ = 53.2, SD = 8.8) from the WWACP who had completed surgery, chemotherapy and/or radiotherapy for breast, gynaecological or blood cancers within the previous 24 months. Sleep problems were measured using the Pittsburgh Sleep Quality Index (PSQI). Baseline data (i.e. prior to intervention randomisation) were analysed.

**Results:**

Most women (59%) reported clinically significant sleep disturbance (PSQI > 5), 40% reported insufficient sleep duration (< 7 h), 38% self-reported poor sleep quality and 28% reported poor habitual sleep efficiency (sleep efficiency < 75%). Fewer psychological and vasomotor climacteric symptoms, age < 45 years and having a partner were associated with reduced odds (AOR < 1) of sleep problems. Higher levels of pain-related disability, and an intermediate compared to ‘high’ level of education, were associated with increased odds (AOR > 1) of sleep problems.

**Conclusions:**

These findings confirm previous studies that have found a high prevalence of sleep problems in women previously treated for cancer. A range of sociodemographic, climacteric and pain-related factors were associated with sleep problems in this study.

**Implications for Cancer Survivors:**

Targeted interventions to improve sleep quality after cancer treatment should be explored in this population. Predictors identified in this study could inform intervention targeting and development.

**Supplementary Information:**

The online version contains supplementary material available at 10.1007/s11764-023-01346-9.

## Introduction

Women previously treated for cancer frequently report persistent treatment-associated health complaints. These include vasomotor symptoms, pain, cognitive alterations [1], fatigue [2] and sleep disturbance [3–5]. Sleep disturbance is the second most frequently reported symptom after fatigue, regardless of disease stage or status [4]. Sleep–wake disturbance is broadly understood as any disruption to sleep quality, sleep timing and sleep-related daytime functioning. A recent systematic review found the overall prevalence of sleep disturbance in women who had completed treatment for breast cancer to be 40% (95%CI = 0.29–0.52) [6]. Although sleep disturbance is also a common symptom of menopause, research shows that the prevalence of sleep disturbance is higher in women with a previous diagnosis of breast cancer than age-matched women without breast cancer [7].

Sleep is modifiable and thus represents a promising opportunity for intervention. It is critical to identify factors associated with sleep problems among women with a previous diagnosis of cancer to inform the ongoing development and targeting of programs to meet their needs as they recover from diagnosis- and treatment-related impacts to daily life. The literature in this area suggests that the impact of sleep disturbance after treatment is influenced by a wide range of sociodemographic factors [e.g. age, education and menopausal status; 6, 8, 9, 10, 11], diagnosis- and treatment-related factors [e.g. cancer stage, treatment type; 8], post-treatment socio-emotional factors [e.g. anxiety and depression; 7, 8] and treatment-related symptoms [e.g. treatment-induced vasomotor symptoms, pain and fatigue; 6, 7, 10, 12, 13]. However, while literature suggests that alternative aetiologies or pathways, such as emotional trauma, pain, depression or physical symptoms can contribute to post-treatment sleep–wake disturbance during, and after cancer, there is substantial inconsistency in how these predictors of sleep disturbance are identified in women treated for cancer [8, 9, 12]. Additionally, much of the literature has examined sleep using single-item scales or a global score combining different components of sleep. Yet sleep is a multidimensional construct. Further research is warranted to examine the correlates of multiple dimensions of subjectively reported sleep among women with a previous diagnosis of cancer.

The purpose of this paper was to examine the prevalence of sleep problems in women enrolled in the Women’s Wellness after Cancer Program (WWACP). We also aimed to explore whether pain, physical activity, depression, anxiety and climacteric symptoms, which are identified in the literature as associated with sleep disturbance, predicted sleep problems in this sample. In this study, we defined sleep problems using five outcomes: insufficient sleep duration (i.e. < 7 h per night), poor self-reported sleep quality, poor habitual sleep efficiency (i.e. < 75%), frequent sleep disturbance and clinically significant sleep disturbance. Based on previous studies, it was predicted that (1) sleep problems would be highly prevalent and (2) sleep problems would be predicted by the hypothesised sociodemographic and post-treatment socio-emotional and physical factors.

## Methods

### Study design, participants and procedure

This paper used baseline data from the WWACP. The WWACP is a structured 12-week e-health intervention targeting physical activity, good diet, smoking cessation, reduction of alcohol intake and sleep hygiene. The study design, participants and procedures have been explained in full previously [14]. Briefly, participants were 351 women (*M*_age_ = 53.2, SD = 8.8; intervention *n* = 175, control group *n* = 176) recruited from major hospital sites in Australia and through partner organisations and consumer groups. Women were eligible to participate if they were aged ≥ 18 years and had completed surgery, chemotherapy and/or radiotherapy for breast, gynaecological or blood cancers within the previous 24 months. The data for this paper were collected prior to randomisation (i.e. baseline) to the intervention or control group for the broader randomised control trial of the WWACP. Participants were excluded if they had metastatic or advanced cancer, inoperable or active locoregional disease or were undertaking maintenance chemotherapy for blood cancers.

This study was conducted according to the guidelines of the Declaration of Helsinki and approved by Queensland University of Technology Human Research Ethics Committee (Approval No: 1300000335). All participants provided written informed consent to participate in the trial.

## Measures

### Sleep problems

The Pittsburgh Sleep Quality Index [PSQI; 15] is a 19-item self-rated measure. The scale has good psychometric properties [15] and is commonly used in studies of cancer patients [16–18]. Respondents rate items based on their usual sleep habits over the last month. The scale comprises seven subscales (or ‘components’): subjective sleep quality, (2) sleep latency, (3) sleep duration, (4) habitual sleep efficiency, (5) sleep disturbances, (6) use of sleep medication and (7) daytime dysfunction. Component scores range from 0 to 3. A global score is also derived based on the sum of all component scores. Higher scores on the component and global scores indicate more severe sleep difficulty. Consistent with other studies [9, 19], we use a cutoff score of > 5 for the global score to indicate clinically significant sleep problems. Other sleep outcomes derived from this measure included insufficient sleep duration (< 7 h, i.e. component score > 0); poor sleep quality (‘fairly bad’ and ‘very bad’ sleep quality, i.e. component score of ≥ 2); poor sleep efficiency (habitual sleep efficiency of ‘65–74%’ and ‘ < 65%, i.e. component score ≥ 2) and frequent sleep disturbance (‘once or twice a week’ and ‘three or more times a week’, i.e. component score ≥ 2).

### Covariates

#### Sociodemographic and clinical data

The following demographic and clinical data were collected: age in years (< 45 years, ≥ 45 years; these categories were used as 45 years of age is commonly used to denote the commencement of perimenopause), BMI (derived from self-reported weight and height: obese/not obese), education level (by category of attainment: low =  ≤ grade 10, intermediate = grade 12, technical or diploma qualification; high = university of postgraduate qualification), marital status (married or de facto, otherwise), income (< $AU20,000 [low], $AU20,000–$80,000 [middle], > $AU80,000 [high]), menopausal status (pre-menopausal, peri-menopausal and post-menopausal).

#### Pain

Pain was measured using the Bodily Pain subscale of the SF-36. Two items comprise the subscale. Items were ‘How much bodily pain have you had during the past 4 weeks?’ and ‘During the past 4 weeks, how much did pain interfere with your normal work (including both work outside the home and housework). The subscale was scored as per standard scoring instructions. This bodily pain subscale has been used as a stand-alone subscale in a cancer population previously [20]. Higher scores indicate less pain-related disability. This subscale was transformed into three categories, each containing a third of the study participants: 1st tertile (0–62), 2nd tertile (63–74) and 3rd tertile (75–100).

#### Climacteric symptoms

Climacteric symptoms were measured with the Greene Climacteric Scale [GCS; 21]. The GCS has 21 self-rated items that assess vasomotor, somatic and psychological symptoms. Participants were asked about the extent to which they were bothered ‘at the moment’ by any of the 21 listed symptoms. Each item was rated on a scale of 0 to 3 (0 = not at all, 1 = a little, 2 = quite a bit, 3 = extremely). The scale has three main scales: psychological (11 items), physical (7 items) and vasomotor (2 items: hot flushes, sweating at night), with an additional item that elicits information about ‘loss of interest in sex’. Higher scores indicate greater severity of the symptoms.

#### Other covariates

Physical activity was measured using the 7-item International Physical Activity Questionnaire Short Form [IPAQ-SF; 22], low ≤ 600 MET.min/wk), moderate = 600–1199 MET.min/wk, high = 1200 + MET.min/wk). Depression-like symptoms were measured using the 20-item Center for Epidemiologic Studies Depression Scale [CES-D; 23], cut score > 16 = risk for clinical depression, and anxiety symptoms were measured with the 20-item Zung Self-rating Anxiety Scale [SAS; 24], cut score > 44 = mild to moderate anxiety levels or greater.

### Data analysis

The proportion of women with insufficient sleep duration, poor sleep quality, poor sleep efficiency, frequent sleep disturbance and clinically significant sleep disturbance was compared across different subgroups by sociodemographic, health and behaviour-related variables. The predictors of the five binary study outcomes were assessed by logistic regression analyses. First, we examined the independent associations between all possible predictors meeting our criteria for inclusion and the five study outcomes (see Supplementary Table 1). There was a high degree of multicollinearity between the CES-D score and the Greene Climacteric Scale total scores, and the Zung self-rating anxiety score and the Greene Climacteric total scores. As a result, the CES-D and Zung variables were not included in subsequent model analyses (see Supplementary Table 2 for descriptive statistics of these variables). Due to substantial amount of missing data on the IPAQ-SF (> 20%), this variable was also removed for subsequent model analyses.

Redundant predictors in a regression model can yield an increase in the log-likelihood and less biased predictions, but they could increase the variance of predictions [25]. Hence, we used the STATA command *gvselect* to identify the best possible subset of the predictors. In this method, the leaps-and-bounds algorithm [26] was applied using the log-likelihoods of candidate models. The best model with the subset predictors was decided based on Akaike’s information criterion (AIC) and Bayesian information criterion (BIC).

### Missing data

There was minimal missing data on the sleep duration (1.8%), sleep quality (1.8%) and sleep efficiency (6.0%) component score outcomes, but substantial missing data on the sleep disturbance component of the PSQI (45%) and consequently the global PSQI score (53.6%). Items were randomly missing throughout the measures, rather than the full measures being skipped. Covariates with > 20% missing data (i.e. the IPAQ-SF) were excluded from the main analysis. Due to the substantial missing values on the sleep disturbance component (and consequently the global PSQI score) of the PSQI, three select PSQI component scores with minimal missing data are reported separately as outcomes (rather than the global score alone). Additionally, all the regression analyses were conducted in three different samples: (1) available sample (unadjusted model), (2) complete sample (adjusted model on complete sample after excluding missing values at covariates and (3) imputed full sample (complete sample after imputing missing values). We used multiple imputations (MI) statistical techniques to impute the missing value in our sample. In MI, the distribution of observed data is used to estimate a set of plausible values for missing data. The missing values are replaced by the estimated plausible values to create a ‘complete’ dataset. In this study, there were various types of covariates that needed to be imputed, such as binary, ordinal and continuous. To impute those types of covariates, we used chained equations, a sequence of univariate imputation methods with fully conditional specification (FCS) of prediction equations using STATA command *mi impute chained*. STATA was used to impute the missing outcome values, which fills in missing values of the variables using a specified regression model in the imputation method. The imputation routine consisted of 1000 iterations to create 30 imputed data sets. Imputations were validated by comparing distributions of covariates before and after imputation. To assess the accuracy of the imputation, several parameters were also examined, such as RVI (relative increase in variance), FMI (fraction of missing information), DF (degrees of freedom), RE (relative efficiency) and the between-imputation and the within-imputation variance estimates. Estimates from the imputed sample were compared with the estimates from the complete case analysis. The results that report the analyses using complete cases (i.e. participants with data on all predictor variables and the sleep outcome) are presented in Supplementary Table 3. We did not observe significant differences in the estimates between the imputed sample and the complete case sample. Therefore, we report estimates from the imputed sample in the main text.

## Results

The background characteristics of the sample are reported in Table 1. Table 1 shows that most participants had a previous diagnosis of breast cancer (95%) and that the average age of the sample was 53 years (SD = 8.8). Although most participants were born in Australia (70%), a portion was born elsewhere (30%). Only 2 participants identified as being Aboriginal or Torres Strait Islander.

**Table 1:** Descriptive statistics and prevalence of sleep problems of participants at baseline

	% (*n*)
No. of cancer-treated women recruited	351
Cancer type; *N* = 284	
- Breast	94.7 (269)
- Other (blood, gynaecological)	5.3 (15)
Mean age (SD)	53.18 (8.77)
Residing states; *N* = 348	
- New South Wales	25.4 (89)
- Victoria	22.2 (78)
- Queensland	25.6 (90)
- South Australia	10.5 (37)
- Western Australia	9.7 (34)
- Tasmania	4.6 (16)
- Australian Capital Territory	2.0 (7)
Country of birth; *N* = 347	
- Australia	69.7 (242)
- Elsewhere	30.3 (105)
Identifies as Aboriginal and/or Torres Strait Islander; 345	
- Yes	0.6 (2)
- No	99.4 (343)
Language other than English; *N* = 345	
- Yes	10.4 (36)
- No	89.6 (309)
Marital status; *N* = 346	
- Married or de facto	76.9 (266)
- Else	23.1 (80)
Employment status; *N* = 346	
- Employed	76.9 (266)
- Else	23.1 (80)
Education; *N* = 346	
- Low	9.0 (31)
- Intermediate	33.5 (116)
- High	57.5 (199)
Income; *N* = 329	
- Less than $20,000 AUD	1.8 (6)
- $20,000–$80,000 AUD	30.4 (100)
- Above $80,000 AUD	67.8 (223)
Sleep problems	
- Insufficient sleep duration; *N* = 345	39.5 (136)
- Poor sleep quality; *N* = 345	38.4 (132)
- Poor sleep efficiency; *N* = 330	28.3 (93)
- Frequent sleep disturbance; *N* = 193	26.4 (51)
- Clinically significant sleep disturbance; *N* = 163	58.9 (93)
PSQI Sleep Medication use component score; *N* = 345	
- Score 0 (not during the past month)	71.0 (245)
- Score 1 (less than once a week)	10.7 (37)
- Scores 2 and 3 (more than once per week)	18.2 (63)
PSQI Daytime dysfunction component score; *N* = 335	
- Score 0 (no difficulty)	20 (67)
- Score 1	60.6 (203)
- Scores 2 and 3 (severe difficulty)	19.4 (65)
PSQI Sleep Latency component score; *N* = 338	
- Score 0 (no difficulty)	18.9 (64)
- Score 1	41.1 (139)
- Score 2 and 3 (severe difficulty)	40 (135)

Insufficient sleep duration is defined as < 7 h per night (sleep duration component score > 0). Poor sleep quality is defined as PSQI subjective sleep quality component score of ≥ 2 (i.e. ‘fairly bad’ and ‘very bad’ sleep quality). Poor sleep efficiency is defined as PSQI ‘habitual sleep efficiency’ score of ≥ 2 (i.e. ‘65–74%’ and ‘ < 65%’). Frequent sleep disturbance is defined as sleep disturbance component score ≥ 2. Clinically significant sleep disturbance is defined as PSQI global score > 5

*PSQI* Pittsburgh Sleep Quality Index

### Prevalence of sleep problems at baseline

**Fig. 1:**
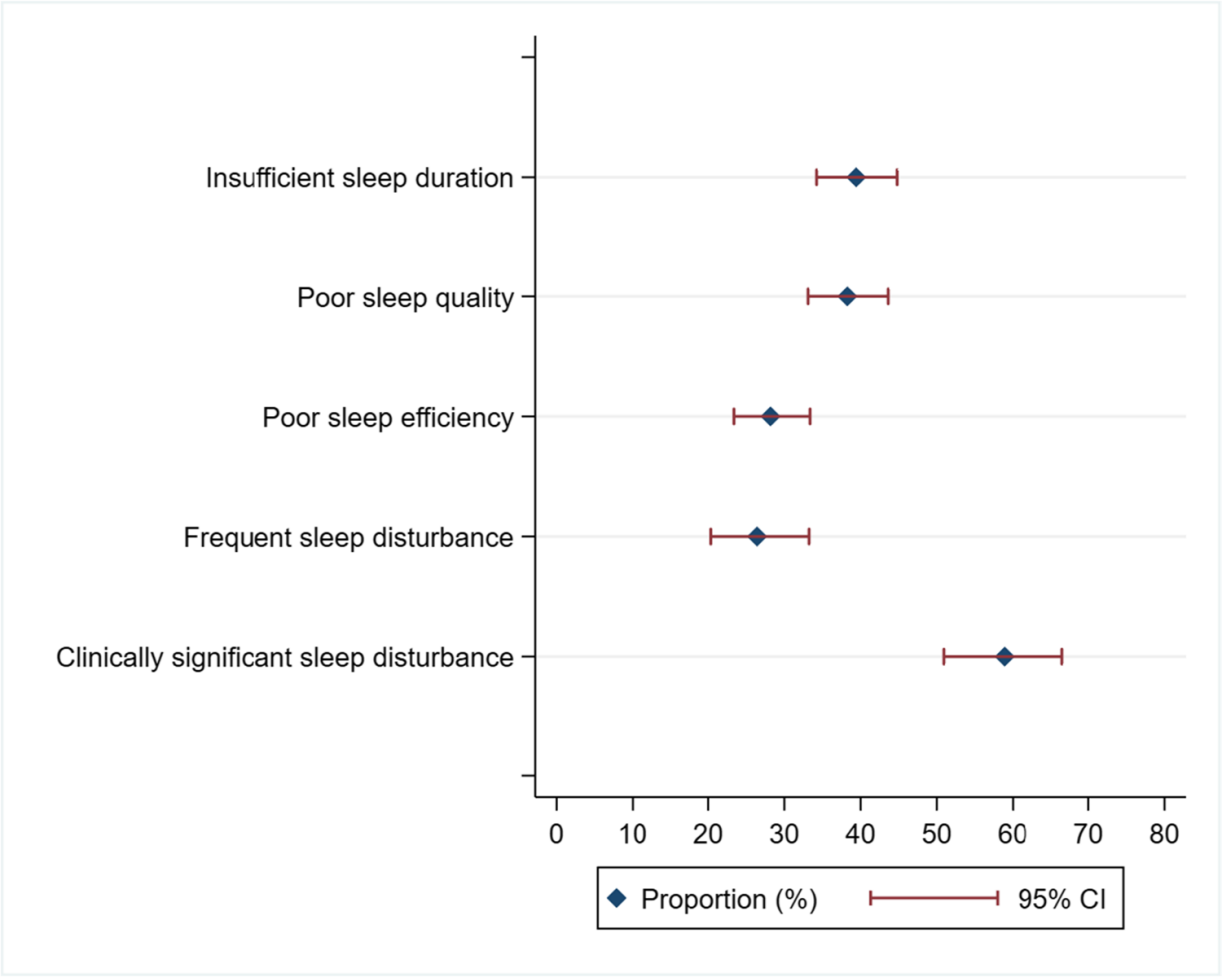
Proportion of sleep-related indicators among cancer-treated women in Australia at baseline

At baseline, 40% of participants reported insufficient sleep duration (i.e. < 7 h sleep per night); 38% reported that their sleep quality was ‘fairly bad’ or ‘very bad’; 28% reported poor habitual sleep efficiency (i.e. 65–74%’ and ‘ < 65%’) and 26% reported frequent sleep disturbances. Based on the total global score (which is an aggregate of all the component scores), 59% of participants exceeded the cut score of 5, a level indicating clinically significant sleep disturbance (see Table 1 and Fig. 1).

### Predictors of sleep problems

Table 2 reports the prevalence of sleep problems among participants with specific clinical, physical or psychological characteristics. Results showing the independent associations between all possible predictors meeting our criteria for inclusion (see data analysis section) and the five study outcomes (having mutually adjusted by all the studied factors) are presented in supplementary Table 1.

**Table 2:** Proportion of participants with sleep problems across different groups of participant characteristics among cancer-treated women

	Sleep indicators; % (group-specific *N*)				
Covariates	Insufficient sleep duration; *N* = 345	Poor sleep quality; *N* = 345	Poor sleep efficiency; *N* = 330	Frequent sleep disturbance; *N* = 193	Clinically significant sleep disturbance; *N* = 163
Age					
- < 45 year	39.7 (116)	37.9 (116)	29.09 (110)	12.7 (63)	57.1 (56)
- ≥ 45 years	39.5 (228)	38.6 (228)	27.85 (219)	33.08 (130)	59.8 (107)
- Missing	0 (1)	0 (1)	0(1)	0(0)	0 (0)
Country of birth					
- Australia	38.3 (240)	38.2 (238)	29.69 (229)	29.55 (132)	62.0 (113)
- Elsewhere	42.7 (103)	39.1 (105)	25.25 (99)	19.67 (61)	52.0 (50)
- Missing	0 (2)	0 (2)	0(2)	0(0)	0 (0)
Marital status					
- Married or de facto	39.8 (264)	38.9 (262)	28.17 (252)	24.16 (178)	58.6 (152)
- Else	39.2 (79)	36.3 (80)	28.95 (76)	57.14 (14)	63.6 (11)
- Missing	0 (2)	33.3 (3)	0 (2)	0 (1)	0 (0)
Employment status					
- Else	42.2 (45)	41.9 (43)	27.91 (43)	46.15 (26)	73.9 (23)
- Employed	39.1 (274)	36.7 (275)	27 (263)	23.84 (151)	54.4 (125)
- Missing	38.5 (26)	48.2 (27)	41.67 (24)	18.75 (16)	73.3 (15)
Education					
- Low	43.3 (30)	38.7 (31)	41.38 (29)	38.89 (18)	66.7 (15)
- Intermediate	51.3 (115)	43.0 (114)	36.36 (110)	32.84 (67)	61.4 (57)
- High	32.0 (197)	35.5 (197)	21.81 (188)	20.37 (108)	56.0 (91)
- Missing	33.3 (3)	33.3 (3)	0 (3)	0 (0)	0 (0)
Income					
- Less than $20,000	33.3 (6)	16.7 (6)	50 (6)	33.33(3)	33.3 (3)
- $20,000–$80,000	41.2 (97)	38.0 (100)	25.81 (93)	26.42(53)	65.1 (43)
- Above $80,000	39.2 (222)	39.8 (221)	28.17 (213)	26.56 (128)	56.3 (112)
- Missing	35.0 (20)	27.8 (18)	33.33 (18)	22.22 (9)	80.0 (5)
Self-reported BMI					
- Not obese	35.5 (234)	36.9 (233)	26.6 (222)	22.73 (132)	55.9 (111)
- Obese	47.7 (88)	41.6 (89)	30.2 (86)	36 (50)	69.1 (42)
- Missing	47.8 (23)	39.1 (23)	36.4 (22)	27.27 (11)	50.0 (10)
Menopausal status					
- Pre-menopausal	21.7 (23)	30.4 (23)	22.7 (22)	23.53 (17)	73.3 (15)
- Peri-menopausal	41.3 (46)	39.1 (46)	27.3 (44)	12.5 (24)	45.5 (22)
- Post-menopausal	40.6 (276)	38.8 (276)	28.8 (264)	28.95 (152)	59.5 (126)
- Missing	0 (0)	0 (0)	0 (0)	0 (0)	0 (0)
Greene subscale: psychological					
- 1st tertile (0–5)	26.1 (115)	14.8 (115)	9.3 (108)	13.64 (66)	33.3 (57)
- 2nd tertile (6–9)	37.3 (110)	36.7 (109)	27.9 (104)	21.43 (56)	57.8 (45)
- 3rd tertile (10–28)	57.3 (103)	62.9 (105)	46.6 (103)	46.67 (60)	87.0 (54)
- Missing	35.3 (17)	56.3 (16)	40.0 (15)	18.18 (11)	57.1 (7)
Greene subscale: vasomotor					
-≤ 2	29.1 (175)	28.6 (175)	19.2 (167)	16.48 (91)	47.4 (76)
- 3–6	50.9 (161)	48.5 (161)	37.0 (154)	35.71 (98)	69.9 (83)
- Missing	33.3 (9)	44.4 (9)	44.4 (9)	25.0 (4)	50.0 (4)
Greene subscale: somatic					
- 1st tertile (0–2)	32.9 (152)	28.5 (151)	20.7 (145)	11.4 (88)	43.1 (72)
- 2nd tertile (4–5)	37.0 (73)	36.8 (76)	23.9 (67)	31.6 (38)	59.4 (32)
- 3rd tertile (6–19)	48.5 (101)	52.5 (99)	38.4 (99)	45.8 (59)	80.8 (52)
- Missing	52.6 (19)	47.4 (19)	47.4 (19)	25.0 (8)	57.1 (7)
Greene subscale: sexual dysfunction					
- No or a little	35.4 (181)	34.8 (181)	24.9 (173)	19.4 (103)	50.6 (87)
- Else	44.9 (147)	42.9 (147)	32.9 (140)	33.7 (83)	68.6 (70)
- Missing	35.3 (17)	35.3 (17)	23.5 (17)	42.9 (7)	66.7 (6)
Pain					
- 1st tertile (0–62)	44.7 (179)	49.2 (179)	34.7 (170)	34.7 (95)	74.1 (81)
- 2nd tertile (63–74)	44.4 (63)	33.3 (63)	23.3 (60)	23.7 (38)	62.5 (32)
- 3rd tertile (75–100)	27.2 (103)	22.3 (103)	20.0 (100)	15.0 (60)	32.0 (50)
- Missing	0 (0)	0 (0)	0 (0)	0 (0)	0 (0)

Insufficient sleep duration is defined as < 7 h per night (sleep duration component score > 0). Poor sleep quality is defined as PSQI subjective sleep quality component score of ≥ 2 (i.e. ‘fairly bad’ and ‘very bad’ sleep quality). Poor sleep efficiency is defined as PSQI ‘habitual sleep efficiency’ score of ≥ 2 (i.e. ‘65–74%’ and ‘ < 65%’). Frequent sleep disturbance is defined as sleep disturbance component score ≥ 2. Clinically significant sleep disturbance is defined as PSQI global score > 5

Taken together, these tables show that the prevalence of sleep problems was significantly associated with a range of hypothesised demographic, clinical, physical or psychological characteristics of cancer-treated women. Of note, the GCS psychological and vasomotor subscales were significantly positively associated with all of the sleep outcomes, such that fewer participants reported poor sleep (i.e. insufficient sleep, poor sleep quality, poor sleep efficiency, frequent sleep disturbances and exceeded the clinical cut off score for the PSQI global score) in the lower tertiles of the GCS vasomotor and psychological subscales compared to the higher tertile, with lower scores on the GCS psychological and vasomotor subscales indicated experiencing fewer of these symptoms. The somatic subscale of the GCS was significantly positively associated with all the sleep outcomes when comparing the first tertile (i.e. lowest somatic symptoms) to the 3rd tertile (i.e. ref; greatest somatic symptoms). When comparing the 2nd tertile (i.e. moderate somatic symptoms) to the 3rd tertile, there were significant positive associations with poor sleep quality and those exceeding the clinical cut off score on the PSQI global score only. The SF36 pain subscale was significantly negatively associated with all the sleep outcomes when comparing the first tertile (i.e. greatest pain-related disability) to the 3rd tertile (i.e. ref; lowest pain-related disability). When comparing the 2nd tertile (i.e. moderate pain-related disability) to the 3rd tertile, there were significant negative associations with insufficient sleep duration and those exceeding the clinical cutoff score on the PSQI global score only. There were other significant bivariate relationships found, although these were less consistently observed across all sleep outcomes. These less consistent variables included the GCS sexual dysfunction scale (sleep disturbance and PSQI global score only), BMI (sleep duration only), education (sleep duration and sleep efficiency only), employment status (sleep disturbance only), marital status (sleep disturbance only) and age (sleep disturbance only).

Table 3 presents the predictors of sleep problems using the best subsets regression approach, which tests all possible combinations of predictor variables, before selecting the best model to fit the data. In the adjusted analysis, the best predictive subset model identified several significant predictors of sleep problems in this study. The most common predictors of sleep problems were psychological and vasomotor symptoms.

**Table 3:** Predictors of sleep-related indicators among cancer-treated women (best predictive subset model)

	OR (95% CI)	
Predictors	Unadjusted	Adjusted*
**Insufficient sleep duration**		
Education		
- Low	1.63 (0.74, 3.55)	1.52 (0.66, 3.49)
- Intermediate	**2.24 (1.4, 3.59)**	**2.17 (1.32, 3.57)**
- High	Ref	Ref
Greene subscale: psychological		
- 1st tertile (0–5)	**0.26 (0.15, 0.47)**	**0.39 (0.21, 0.72)**
- 2nd tertile (6–9)	**0.44 (0.26, 0.77)**	**0.51 (0.29, 0.89)**
- 3rd tertile (10–28)	Ref	Ref
Greene subscale: vasomotor		
-≤ 2	**0.40 (0.25, 0.62)**	**0.46 (0.29, 0.75)**
- 3–6	Ref	Ref
Pain		
- 1st tertile (0–62)	**2.16 (1.28, 3.66)**	1.72 (0.98, 3.04)
- 2nd tertile (63–74)	**2.14 (1.11, 4.15)**	1.99 (0.99, 4.01)
- 3rd tertile (75–100)	Ref	Ref
**Poor sleep quality**		
Greene subscale: psychological		
- 1st tertile (0–5)	**0.10 (0.05, 0.2)**	**0.14 (0.07, 0.28)**
- 2nd tertile (6–9)	**0.34 (0.2, 0.6)**	**0.35 (0.19, 0.62)**
- 3rd tertile (10–28)	Ref	Ref
Greene subscale: vasomotor		
- ≤ 2	**0.43 (0.27, 0.67)**	**0.59 (0.35, 0.97)**
- 3–6	Ref	Ref
Pain		
- 1st tertile (0–62)	**3.36 (1.94, 5.82)**	**2.42 (1.33, 4.42)**
- 2nd tertile (63–74)	1.74 (0.86, 3.5)	1.43 (0.67, 3.08)
- 3rd tertile (75–100)	Ref	Ref
Poor sleep efficiency		
Marital status		
- Married or de facto	0.96 (0.55, 1.70)	0.88 (0.47, 1.64)
- Single, widowed, separated or divorced	Ref	Ref
Education		
- Low	**2.53 (1.12, 5.72)**	2.41 (0.98, 5.93)
- Intermediate	**2.05 (1.22, 3.45)**	**1.86 (1.06, 3.25)**
- High	Ref	Ref
Greene subscale: psychological		
- 1st tertile (0–5)	**0.12 (0.05, 0.25)**	**0.15 (0.07, 0.32)**
- 2nd tertile (6–9)	**0.44 (0.25, 0.79)**	**0.48 (0.26, 0.86)**
- 3rd tertile (10–28)	Ref	Ref
Greene subscale: vasomotor		
- ≤ 2	**0.40 (0.24, 0.67)**	**0.53 (0.31, 0.92)**
- 3–6	Ref	Ref
**Frequent sleep disturbance**		
Age		
- < 45 year	**0.29 (0.13, 0.67)**	**0.25 (0.10, 0.62)**
- ≥ 45 years	Ref	Ref
Marital status		
- Married or de facto	**0.24 (0.08, 0.73)**	**0.23 (0.06, 0.91)**
- Single, widowed, separated or divorced	Ref	Ref
Greene subscale: psychological		
- 1st tertile (0–5)	**0.18 (0.08, 0.43)**	**0.27 (0.10, 0.70)**
- 2nd tertile (6–9)	**0.31 (0.14, 0.70)**	**0.30 (0.12, 0.72)**
- 3rd tertile (10–28)	Ref	Ref
Greene subscale: vasomotor		
- ≤ 2	**0.36 (0.18, 0.71)**	**0.40 (0.18, 0.88)**
- 3–6	Ref	Ref
Greene subscale: sexual dysfunction		
- No or a little	**0.47 (0.24, 0.92)**	0.52 (0.24, 1.13)
- Else	Ref	Ref
**Clinically significant sleep disturbance**		
Employment status		
- Employed	0.42 (0.16, 1.14)	0.39 (0.12, 1.25)
- Else	Ref	Ref
Greene subscale: psychological		
- 1st tertile (0–5)	**0.07 (0.03, 0.20)**	**0.11 (0.04, 0.30)**
- 2nd tertile (6–9)	**0.20 (0.08, 0.55)**	**0.18 (0.06, 0.54)**
- 3rd tertile (10–28)	Ref	Ref
Greene subscale: vasomotor		
- ≤ 2	**0.39 (0.20, 0.74)**	0.52 (0.23, 1.17)
- 3–6	Ref	Ref
Pain		
- 1st tertile (0–62)	**6.07 (2.80, 13.17)**	**4.87 (1.98, 11.97)**
- 2nd tertile (63–74)	**3.54 (1.40, 8.98)**	**3.38 (1.17, 9.73)**
- 3rd tertile (75–100)	Ref	Ref

^*^Missing imputed analysis. Bolded values are significant. Insufficient sleep duration is defined as < 7 h per night (sleep duration component score > 0). Poor sleep quality is defined as PSQI subjective sleep quality component score of ≥ 2 (i.e. ‘fairly bad’ and ‘very bad’ sleep quality). Poor sleep efficiency is defined as PSQI ‘habitual sleep efficiency’ score of ≥ 2 (i.e. ‘65–74%’ and ‘ < 65%’). Frequent sleep disturbance is defined as sleep disturbance component score ≥ 2. Clinically significant sleep disturbance is defined as PSQI global score > 5

The direction of the effects shows that an intermediate (i.e. grade 12, technical or diploma qualification) compared to higher education was associated with greater odds of insufficient sleep duration and poor sleep efficiency. Fewer psychological and vasomotor symptoms were significantly associated with less odds of sleep problems on all sleep outcomes (except for exceeding the PSQI clinical cutoff score for vasomotor symptoms). Participants with greater pain-related disability had significantly greater odds of poor sleep quality and of exceeding the PSQI clinical cutoff score. Being younger and married or de facto was significantly associated with reduced odds of frequent sleep disturbance (Table 3).

## Discussion

The aim of this analysis was to understand the predictors of sleep problems in women previously treated for cancer. As hypothesised, the prevalence of insufficient sleep duration (40%) and poor sleep quality (38%) was high at baseline. Many participants (59%) exceeded the cut score indicative of clinically significant sleep disturbance on the PSQI. The predictors of sleep problems in this sample included age, education, marital status, psychological symptoms (as measured on the GCS), vasomotor symptoms (as measured on the GCS) and pain.

The prevalence of sleep problems in this sample was high for insufficient sleep duration (40%), poor sleep quality (38%) and clinically significant sleep disturbance (> 5 PSQI global score; 59%). The prevalence estimates were comparable with other studies of women treated for cancer. For example, Colagiuri found that 58% of women who were 3–4 months post-breast cancer surgery reported clinically significant sleep disturbance (PSQI > 5) [9]. Our prevalence estimates were also within the confidence interval for the pooled estimate of sleep disturbance prevalence (i.e. 0.40 95%CI [0.29–0.52]) from a systematic review of the prevalence of sleep disturbance in women who had completed breast cancer treatment [6]; however, the included studies from this review employed various cut scores on the PSQI along with the inclusion of other measures of sleep problems (e.g. Insomnia Severity Index (ISI)) so it is challenging to make comparisons. Our findings suggest that sleep problems are a common problem after treatment. As such, opportunities to improve the management and treatment of sleep problems should be explored in this population.

We identified a range of predictors of sleep problems at the baseline assessment. The sociodemographic factors associated with better sleep outcomes at baseline included age less than 45 years and being married or in a de facto relationship. The literature on the association between age and sleep disturbance among women with cancer is mixed and unresolved [6]. Our result was consistent with another large study of 3343 women recently treated for breast cancer [9], but inconsistent with a recent study that reported that younger age was a risk factor for sleep disturbance in 632 African American women treated for breast cancer 24 months post-diagnosis [8], and another study that found no association between age and sleep quality in 246 women treated for breast cancer 1–10 years post-treatment [12]. Our marital status finding was consistent with broader general population studies of sleep and marital relationships in which never married or previously married men and women reported more frequent insufficient sleep or sleep disturbances than their married counterparts [27, 28], and married older adults had better objectively measured sleep than unmarried older adults [29]. Finally, having an intermediate compared to high level of education was associated with increased risk of insufficient sleep duration and poor sleep efficiency. Less education was also identified as a risk factor for sleep disturbance or insufficient sleep duration in other studies [8, 10]. However, other studies have found that higher education was a risk factor for insomnia [11] and greater sleep disturbance over time [30] among women who had been treated for breast cancer. Our findings suggest that sociodemographic factors could predict sleep problems after breast cancer treatment in Australian women. These findings could be used to better target sleep interventions in this population.

Higher levels of pain-related disability were associated with an increased risk of poor sleep quality and clinically significant sleep disturbance (PSQI > 5). Pain has been previously identified as a predictor of poor sleep in cancer-treated patients [10, 31]. Pain is a well-known risk factor for insomnia in non-cancer populations also [32], and it has been associated with insufficient sleep in the general population [33]. It could be that, in WWACP participants, pain interfered with their sleep quality. However, due to the study design, we were unable to determine whether the relationship between pain and poor sleep was causal. Some research has shown that the relationship between pain and sleep is bidirectional [34]. It has been suggested that disturbed sleep and related fatigue might lower the coping threshold for pain symptoms [31]. More research using longitudinal data is needed to further examine these issues. Untangling these issues will better inform the management of pain in this population, which could include improved management of analgesia, sleep or both [31].

Consistent with other studies [35–37], we found that climacteric symptoms, particularly psychological and vasomotor symptoms, were associated with insufficient sleep, poor sleep efficiency and poorer self-reported sleep quality. Hot flashes and night sweats in particular have been shown to predict poor sleep quality in patients undergoing treatment [37] and throughout the recovery trajectory [12]. However, not all studies have observed a relationship between vasomotor symptoms and sleep [38]. Menopausal hot flashes are reported as more frequent, severe, distressing and of greater duration in breast cancer-treated women than in similarly aged healthy women [39–41]. Although our study design did not identify the temporal relationship between hot flashes and night sweats, and sleep (i.e. whether the symptoms were experienced nocturnally or not), previous research by Savard and colleagues [37] observed that more wake time and lighter sleep (objectively measured) were more likely to occur within 10 min of a nocturnal hot flash (again, objectively measured) than at other times in women who have had breast cancer. Poor concordance between objectively and subjectively reported hot flashes has been observed during sleep compared to waking-hours, with more hot flashes recorded using skin conductance monitoring [42]. Consequently, the reported association between hot flashes and night sweats, and sleep in this study might in fact underestimate the association. Overall, our findings further support vasomotor symptoms as a significant contributor to sleep outcomes in women after cancer treatment. Given our finding that hot flashes and night sweats are associated with a range of sleep outcomes, interventions that target these symptoms are indicated. Future iterations of the WWACP will more directly target vasomotor symptoms to improve this aspect of sleep.

This study has several limitations. The results of this study might not generalise to other cancer populations. Second, sleep was measured using a self-report measure only, which is subject to self-report bias. Although our use of a standardised measure improves on previous research, which has tended to rely on single-item measures of the construct, future studies should consider conducting more comprehensive measurement of this construct. For example, objective measurement of sleep (e.g. via actigraphy or polysomnography) can provide important information about the physiological features of sleep that is not possible with self-report. Future studies could also examine sleep in context, such as collecting information about the sleep environment, bed partner and work schedules (e.g. shift work) that could influence sleep disturbance after treatment for cancer. Another limitation of this study was that there was no pre-diagnosis estimate or pre-treatment measure of sleep disturbance. Previous research shows women report high levels of sleep disturbance prior to treatment of their breast cancer [43, 44]. Also, sleep disturbance, and some sleep disorders such as obstructive sleep apnoea, can be risk factors for breast cancer [45, 46], although more research is needed. The PSQI and other covariates also had substantial missing data, likely due to the large battery of questionnaires that the women completed without being prompted to respond to each missed item. Although we used statistical techniques to impute these data, future research should take care to follow up on missing responses. Furthermore, we did not examine medications in this study. It is possible that some women were taking medications/hormone therapy that may affect sleep. Finally, our study reports associations between study variables and sleep outcomes. As such, causal interpretations should not be drawn from these findings.

In conclusion, women experiencing disability and consequences from cancer diagnosis and treatment have diverse and ongoing care or psychosocial health needs, including sleep problems. As such, the findings from this study indicate the need for the development and implementation of improved sleep interventions for women who have previously been treated for cancer to support them. These interventions should account for the psychological and physical symptoms experienced by this population. Managing sleep after treatment for cancer represents an important intervention and general health promotion opportunity.

### Supplementary Information

Below is the link to the electronic supplementary material.

Supplementary file1 (DOCX 48 KB)

Supplementary file2 (DOCX 19 KB)

Supplementary file3 (DOCX 28 KB)

## Data Availability

The datasets generated during and/or analysed during the current study are available from the corresponding author on reasonable request.

## References

[1] Evens K, Eschiti VS (2009). Cognitive effects of cancer treatment: “Chemo Brain” Explained. Clin J Oncol Nursing.

[2] Bower JE (2006). Fatigue in long-term breast carcinoma survivors: a longitudinal investigation. Cancer.

[3] Costa AR (2014). Impact of breast cancer treatments on sleep disturbances–a systematic review. Breast.

[4] Cleeland CS (2013). The symptom burden of cancer: evidence for a core set of cancer-related and treatment-related symptoms from the Eastern Cooperative Oncology Group Symptom Outcomes and Practice Patterns study. Cancer.

[5] Janz NK (2007). Symptom experience and quality of life of women following breast cancer treatment. J Women’s Health.

[6] Leysen L (2019). Prevalence and risk factors of sleep disturbances in breast cancersurvivors: systematic review and meta-analyses. Support Care Cancer.

[7] Otte JL (2010). Prevalence, severity, and correlates of sleep-wake disturbances in long-term breast cancer survivors. J Pain Symptom Manage.

[8] Gonzalez BD (2021). Prevalence, risk factors, and trajectories of sleep disturbance in a cohort of African-American breast cancer survivors. Support Care Cancer.

[9] Colagiuri B (2011). Prevalence and predictors of sleep difficulty in a national cohort of women with primary breast cancer three to four months postsurgery. J Pain Symptom Manage.

[10] Klyushnenkova EN, Sorkin JD, Gallicchio L (2015). Association of obesity and sleep problems among breast cancer survivors: results from a registry-based survey study. Support Care Cancer.

[11] Savard J (2001). Prevalence, clinical characteristics, and risk factors for insomnia in the context of breast cancer. Sleep.

[12] Lowery-Allison AE (2018). Sleep problems in breast cancer survivors 1–10 years posttreatment. Palliat Support Care.

[13] Bower JE (2000). Fatigue in breast cancer survivors: occurrence, correlates, and impact on quality of life. J Clin Oncol.

[14] Anderson D (2017). The Women’s wellness after cancer program: a multisite, single-blinded, randomised controlled trial protocol. BMC Cancer.

[15] Buysse DJ (1989). The Pittsburgh Sleep Quality Index: a new instrument for psychiatric practice and research. Psychiatry Res.

[16] Carpenter JS, Andrykowski MA (1998). Psychometric evaluation of the Pittsburgh sleep quality index. J Psychosom Res.

[17] Beck SL (2004). Psychometric evaluation of the Pittsburgh Sleep Quality Index in cancer patients. J Pain Symptom Manage.

[18] Otte JL (2015). Systematic review of sleep disorders in cancer patients: can the prevalence of sleep disorders be ascertained?. Cancer Med.

[19] Buysse DJ (2008). Relationships between the Pittsburgh Sleep Quality Index (PSQI), Epworth Sleepiness Scale (ESS), and clinical/polysomnographic measures in a community sample. J Clin Sleep Med.

[20] Forsythe LP (2013). Pain in long-term breast cancer survivors: the role of body mass index, physical activity, and sedentary behavior. Breast Cancer Res Treat.

[21] Greene JG (2008). Constructing a standard climacteric scale. Maturitas.

[22] Craig CL (2003). International physical activity questionnaire: 12-country reliability and validity. Med Sci Sports Exerc.

[23] Radloff LS (1977). The CES-D scale: A self-report depression scale for research in the general population. Appl Psychol Meas.

[24] Zung WW (1971). A rating instrument for anxiety disorders. Psychosom: J Consult Liaison Psychiatry.

[25] Lindsey C, Sheather S (2015). Best subsets variable selection in nonnormal regression models. Stand Genomic Sci.

[26] Furnival GM, Wilson RW (2000). Regressions by leaps and bounds. Technometrics.

[27] Chapman DP (2012). Household demographics and perceived insufficient sleep among US adults. J Community Health.

[28] Arber S, Bote M, Meadows R (2009). Gender and socio-economic patterning of self-reported sleep problems in Britain. Soc Sci Med.

[29] Chen J-H, Waite LJ, Lauderdale DS (2015). Marriage, relationship quality, and sleep among US older adults. J Health Soc Behav.

[30] Van Onselen C (2013). Trajectories of sleep disturbance and daytime sleepiness in women before and after surgery for breast cancer. J Pain Symptom Manage.

[31] Koopman C (2002). Sleep disturbances in women with metastatic breast cancer. Breast J.

[32] Whibley D (2019). Sleep and pain: a systematic review of studies of mediation. Clin J Pain.

[33] Strine TW, Chapman DP (2005). Associations of frequent sleep insufficiency with health-related quality of life and health behaviors. Sleep Med.

[34] Haack M (2020). Sleep deficiency and chronic pain: potential underlying mechanisms and clinical implications. Neuropsychopharmacol.

[35] Stein KD (2000). Impact of hot flashes on quality of life among postmenopausal women being treated for breast cancer. J Pain Symptom Manag.

[36] Couzi RJ, Helzlsouer KJ, Fetting JH (1995). Prevalence of menopausal symptoms among women with a history of breast cancer and attitudes toward estrogen replacement therapy. J Clin Oncol.

[37] Savard J (2004). The association between nocturnal hot flashes and sleep in breast cancer survivors. J Pain Symptom Manag.

[38] Alfano CM (2011). Sleep duration change across breast cancer survivorship: associations with symptoms and health-related quality of life. Breast Cancer Res Treat.

[39] Carpenter JS, Johnson D, Wagner L, Andrykowski M (2002). Hot flashes and related outcomes in breast cancer survivors and matched comparison women. Oncol Nurs Forum..

[40] Seib C (2017). Menopausal symptom clusters and their correlates in women with and without a history of breast cancer: a pooled data analysis from the Women’s Wellness Research Program. Menopause.

[41] Gupta P (2006). Menopausal symptoms in women treated for breast cancer: the prevalence and severity of symptoms and their perceived effects on quality of life. Climacteric.

[42] Carpenter JS (1999). Feasibility and psychometrics of an ambulatory hot flash monitoring device. Menopause.

[43] Ancoli-Israel S (2006). Fatigue, sleep, and circadian rhythms prior to chemotherapy for breast cancer. Support Care Cancer.

[44] Denieffe S, Cowman S, Gooney M (2014). Symptoms, clusters and quality of life prior to surgery for breast cancer. J Clin Nurs.

[45] Mogavero MP (2021). Sleep disorders and cancer: State of the art and future perspectives. Sleep Med Rev.

[46] Choi JH (2019). Association between obstructive sleep apnoea and breast cancer: the Korean National Health Insurance Service Data 2007–2014. Sci Rep.

