# Supplementary material for: Prevalence and predictors of sleep problems in women following a cancer diagnosis: results from the women’s wellness after cancer program
Source: J Cancer Surviv. 2023 Feb 24;18(3):960–71. doi: 10.1007/s11764-023-01346-9 (PMC11082004; doi:10.1007/s11764-023-01346-9)
Supplement: Supplementary file 1 — Supplementary file1 (DOCX 48 KB) [file 11764_2023_1346_MOESM1_ESM.docx]

**Supplementary tables**

**Supplementary Table 1: Predictors of sleep related indicators among cancer treated women (All predictors of interest)**

|  | **OR (95%CI)** | | |
| --- | --- | --- | --- |
| **Predictors** | **Unadjusted (available case)** | **Adjusted (Complete case)** | **Adjusted (Missing imputed)** |
| **Insufficient sleep duration** |  |  |  |
| Age |  |  |  |
| -        <45 year | 1.01(0.64, 1.59) | 1.21(0.6, 2.43) | 1.27(0.71, 2.28) |
| -        >= 45 years | Ref | Ref | Ref |
| Country of birth |  |  |  |
| -        Australia | 0.83(0.52, 1.33) | 0.89(0.47, 1.67) | 0.77(0.45, 1.29) |
| -        Elsewhere | Ref | Ref | Ref |
| Marital status |  |  |  |
| -        Married or de facto | 1.02(0.61, 1.71) | 0.67(0.33, 1.37) | 0.94(0.52, 1.68) |
| -        -Else | Ref | Ref | Ref |
| Employment status |  |  |  |
| -        Employed | 0.88(0.46, 1.66) | 0.54(0.22, 1.33) | 0.67(0.3, 1.48) |
| -        Else | Ref | Ref | Ref |
| Education |  |  |  |
| -        Low | 1.63(0.74, 3.55) | 2.38(0.72, 7.88) | 1.59(0.64, 3.94) |
| -        Intermediate | 2.24(1.4, 3.59) | 2.09(1.09, 4.01) | 2.22(1.29, 3.81) |
| -        High | Ref | Ref | Ref |
| Income |  |  |  |
| -        Less than $20,000 | 0.78(0.14, 4.33) | 1(0, 0) | 0.83(0.12, 5.56) |
| -        $20,000 - $80,000 | 1.09(0.67, 1.77) | 0.93(0.47, 1.87) | 0.85(0.47, 1.53) |
| -        Above $80,000 | Ref | Ref | Ref |
| Self-reported BMI |  |  |  |
| -        Obese | 1.66(1.01, 2.73) | 1.34(0.67, 2.68) | 1.3(0.73, 2.31) |
| -        Else | Ref | Ref | Ref |
| Menopausal status |  |  |  |
| -        Pre-menopausal | 0.41(0.15, 1.13) | 0.67(0.15, 2.91) | 0.46(0.15, 1.46) |
| -        Peri menopausal | 1.03(0.55, 1.94) | 2.17(0.88, 5.34) | 1.09(0.51, 2.35) |
| -        Post-menopausal | Ref | Ref | Ref |
| Greene subscale: psychological |  |  |  |
| -        1st tertile (0-5) | 0.26(0.15, 0.47) | 0.49(0.23, 1.08) | 0.43(0.21, 0.86) |
| -        2nd tertile (6-9) | 0.44(0.26, 0.77) | 0.5(0.24, 1.04) | 0.5(0.27, 0.9) |
| -        3rd tertile (10-28) | Ref | Ref | Ref |
| Greene subscale: vasomotor |  |  |  |
| -        <=2 | 0.4(0.25, 0.62) | 0.4(0.22, 0.73) | 0.47(0.28, 0.78) |
| -        3-6 | Ref | Ref | Ref |
| Greene subscale: somatic |  |  |  |
| -        1st tertile (0-2) | 0.52(0.31, 0.87) | 1.01(0.44, 2.3) | 0.98(0.49, 1.97) |
| -        2nd tertile (4-5) | 0.62(0.34, 1.15) | 0.65(0.29, 1.49) | 0.77(0.38, 1.57) |
| -        3rd tertile (6-19) | Ref | Ref | Ref |
| Greene subscale: sexual dysfunction |  |  |  |
| -        no or a little | 0.67(0.43, 1.05) | 0.77(0.42, 1.41) | 0.89(0.53, 1.51) |
| -        else | Ref | Ref | Ref |
| Pain |  |  |  |
| -        1st tertile (0-62) | 2.16(1.28, 3.66) | 2.17(0.98, 4.8) | 1.75(0.92, 3.34) |
| -        2nd tertile (63-74) | 2.14(1.11, 4.15) | 1.67(0.68, 4.12) | 1.98(0.95, 4.14) |
| -        3rd tertile (75-100) | Ref | Ref | Ref |
| **Poor sleep quality** |  |  |  |
| Age |  |  |  |
| -        <45 year | 0.97(0.61, 1.54) | 0.86(0.41, 1.83) | 1.09(0.59, 2.01) |
| -        >= 45 years | Ref | Ref | Ref |
| Country of birth |  |  |  |
| -        Australia | 0.97(0.6, 1.55) | 0.85(0.44, 1.65) | 0.94(0.54, 1.61) |
| -        Elsewhere | Ref | Ref | Ref |
| Marital status |  |  |  |
| -        Married or de facto | 1.12(0.67, 1.88) | 0.82(0.39, 1.74) | 0.98(0.53, 1.83) |
| -        -Else | Ref | Ref | Ref |
| Employment status |  |  |  |
| -        Employed | 0.81(0.42, 1.55) | 0.57(0.22, 1.45) | 0.6(0.27, 1.34) |
| -        Else | Ref | Ref | Ref |
| Education |  |  |  |
| -        Low | 1.15(0.53, 2.5) | 0.85(0.21, 3.43) | 1.07(0.4, 2.83) |
| -        Intermediate | 1.37(0.85, 2.19) | 1.12(0.57, 2.22) | 1.29(0.74, 2.26) |
| -        High | Ref | Ref | Ref |
| Income |  |  |  |
| -        Less than $20,000 | 0.3(0.03, 2.63) | 1(0, 0) | 0.32(0.03, 3.58) |
| -        $20,000 - $80,000 | 0.93(0.57, 1.51) | 0.82(0.4, 1.7) | 0.66(0.36, 1.22) |
| -        Above $80,000 | Ref | Ref | Ref |
| Self-reported BMI |  |  |  |
| -        Obese | 1.22(0.74, 2) | 0.85(0.41, 1.77) | 0.9(0.49, 1.65) |
| -        Else | Ref | Ref | Ref |
| Menopausal status |  |  |  |
| -        Pre-menopausal | 0.69(0.28, 1.73) | 0.46(0.08, 2.72) | 0.93(0.3, 2.9) |
| -        Peri menopausal | 1.02(0.54, 1.93) | 1.29(0.5, 3.32) | 0.87(0.39, 1.97) |
| -        Post-menopausal | Ref | Ref | Ref |
| Greene subscale: psychological |  |  |  |
| -        1st tertile (0-5) | 0.1(0.05, 0.2) | 0.16(0.07, 0.37) | 0.14(0.06, 0.29) |
| -        2nd tertile (6-9) | 0.34(0.2, 0.6) | 0.31(0.15, 0.65) | 0.32(0.18, 0.6) |
| -        3rd tertile (10-28) | Ref | Ref | Ref |
| Greene subscale: vasomotor |  |  |  |
| -        <=2 | 0.43(0.27, 0.67) | 0.48(0.25, 0.9) | 0.57(0.34, 0.97) |
| -        3-6 | Ref | Ref | Ref |
| Greene subscale: somatic |  |  |  |
| -        1st tertile (0-2) | 0.36(0.21, 0.61) | 0.94(0.4, 2.23) | 0.98(0.48, 2) |
| -        2nd tertile (4-5) | 0.53(0.29, 0.97) | 0.77(0.33, 1.77) | 0.77(0.38, 1.59) |
| -        3rd tertile (6-19) | Ref | Ref | Ref |
| Greene subscale: sexual dysfunction |  |  |  |
| -        no or a little | 0.71(0.46, 1.11) | 0.96(0.51, 1.82) | 1.1(0.63, 1.9) |
| -        else | Ref | Ref | Ref |
| Pain |  |  |  |
| -        1st tertile (0-62) | 3.36(1.94, 5.82) | 2.7(1.13, 6.43) | 2.52(1.26, 5.02) |
| -        2nd tertile (63-74) | 1.74(0.86, 3.5) | 1.22(0.43, 3.44) | 1.41(0.62, 3.16) |
| -        3rd tertile (75-100) | Ref | Ref | Ref |
| **Poor sleep efficiency** |  |  |  |
| Age |  |  |  |
| -        <45 year | 1.06(0.64, 1.76) | 1.37(0.6, 3.15) | 1.3(0.68, 2.5) |
| -        >= 45 years | Ref | Ref | Ref |
| Country of birth |  |  |  |
| -        Australia | 1.25(0.73, 2.13) | 1.53(0.69, 3.39) | 1.19(0.65, 2.19) |
| -        Elsewhere | Ref | Ref | Ref |
| Marital status |  |  |  |
| -        Married or de facto | 0.96(0.55, 1.7) | 0.39(0.17, 0.9) | 0.81(0.42, 1.59) |
| -        -Else | Ref | Ref | Ref |
| Employment status |  |  |  |
| -        Employed | 0.96(0.47, 1.96) | 0.45(0.15, 1.34) | 0.77(0.32, 1.83) |
| -        Else | Ref | Ref | Ref |
| Education |  |  |  |
| -        Low | 2.53(1.12, 5.72) | 2.61(0.62, 10.89) | 2.78(1.01, 7.68) |
| -        Intermediate | 2.05(1.22, 3.45) | 2.17(1.01, 4.64) | 2.2(1.21, 4.03) |
| -        High | Ref | Ref | Ref |
| Income |  |  |  |
| -        Less than $20,000 | 2.55(0.5, 12.99) | 1(0, 0) | 3.71(0.47, 29.37) |
| -        $20,000 - $80,000 | 0.89(0.51, 1.54) | 0.87(0.37, 2.02) | 0.61(0.31, 1.22) |
| -        Above $80,000 | Ref | Ref | Ref |
| Self-reported BMI |  |  |  |
| -        Obese | 1.2(0.69, 2.07) | 0.74(0.32, 1.7) | 0.83(0.43, 1.61) |
| -        Else | Ref | Ref | Ref |
| Menopausal status |  |  |  |
| -        Pre-menopausal | 0.73(0.26, 2.04) | 0.31(0.03, 3.14) | 0.99(0.28, 3.45) |
| -        Peri menopausal | 0.93(0.45, 1.9) | 1.92(0.68, 5.48) | 0.8(0.33, 1.92) |
| -        Post-menopausal | Ref | Ref | Ref |
| Greene subscale: psychological |  |  |  |
| -        1st tertile (0-5) | 0.12(0.05, 0.25) | 0.11(0.04, 0.34) | 0.13(0.05, 0.33) |
| -        2nd tertile (6-9) | 0.44(0.25, 0.79) | 0.39(0.17, 0.87) | 0.45(0.24, 0.85) |
| -        3rd tertile (10-28) | Ref | Ref | Ref |
| Greene subscale: vasomotor |  |  |  |
| -        <=2 | 0.4(0.24, 0.67) | 0.4(0.19, 0.83) | 0.55(0.31, 0.98) |
| -        3-6 | Ref | Ref | Ref |
| Greene subscale: somatic |  |  |  |
| -        1st tertile (0-2) | 0.42(0.24, 0.74) | 0.96(0.36, 2.57) | 1.03(0.48, 2.23) |
| -        2nd tertile (4-5) | 0.5(0.25, 1.01) | 0.43(0.16, 1.16) | 0.7(0.32, 1.54) |
| -        3rd tertile (6-19) | Ref | Ref | Ref |
| Greene subscale: sexual dysfunction |  |  |  |
| -        no or a little | 0.68(0.41, 1.11) | 1.65(0.79, 3.41) | 1.13(0.63, 2.06) |
| -        else | Ref | Ref | Ref |
| Pain |  |  |  |
| -        1st tertile (0-62) | 2.13(1.19, 3.81) | 1.58(0.6, 4.12) | 1.58(0.76, 3.32) |
| -        2nd tertile (63-74) | 1.22(0.56, 2.64) | 1.06(0.33, 3.36) | 1.04(0.43, 2.55) |
| -        3rd tertile (75-100) | Ref | Ref | Ref |
| **Sleep disturbance** |  |  |  |
| Age |  |  |  |
| -        <45 year | 0.29(0.13, 0.67) | 0.28(0.08, 1.01) | 0.25(0.08, 0.81) |
| -        >= 45 years | Ref | Ref | Ref |
| Country of birth |  |  |  |
| -        Australia | 1.71(0.82, 3.57) | 1.85(0.66, 5.2) | 1.82(0.74, 4.46) |
| -        Elsewhere | Ref | Ref | Ref |
| Marital status |  |  |  |
| -        Married or de facto | 0.24(0.08, 0.73) | 0.1(0.01, 0.69) | 0.2(0.04, 0.97) |
| -        -Else | Ref | Ref | Ref |
| Employment status |  |  |  |
| -        Employed | 0.37(0.15, 0.86) | 0.39(0.1, 1.46) | 0.23(0.07, 0.72) |
| -        Else | Ref | Ref | Ref |
| Education |  |  |  |
| -        Low | 2.49(0.86, 7.16) | 1.5(0.31, 7.11) | 1.58(0.4, 6.2) |
| -        Intermediate | 1.91(0.96, 3.82) | 1.35(0.47, 3.9) | 1.55(0.63, 3.82) |
| -        High (2) | Ref | Ref | Ref |
| Income |  |  |  |
| -        Less than $20,000 | 1.38(0.12, 15.74) | 1(0, 0) | 0.5(0.01, 37.12) |
| -        $20,000 - $80,000 | 0.99(0.48, 2.05) | 0.47(0.15, 1.49) | 0.33(0.12, 0.95) |
| -        Above $80,000 | Ref | Ref | Ref |
| Self-reported BMI |  |  |  |
| -        Obese | 1.91(0.94, 3.88) | 0.6(0.2, 1.83) | 1.22(0.48, 3.07) |
| -        Else | Ref | Ref | Ref |
| Menopausal status |  |  |  |
| -        Pre-menopausal | 0.76(0.23, 2.44) | 0.65(0.06, 7.04) | 2.29(0.42, 12.4) |
| -        Peri menopausal | 0.35(0.1, 1.24) | 0.57(0.1, 3.22) | 0.73(0.14, 3.8) |
| -        Post-menopausal | Ref | Ref | Ref |
| Greene subscale: psychological |  |  |  |
| -        1st tertile (0-5) | 0.18(0.08, 0.43) | 0.31(0.08, 1.2) | 0.34(0.09, 1.21) |
| -        2nd tertile (6-9) | 0.31(0.14, 0.7) | 0.16(0.04, 0.61) | 0.27(0.09, 0.79) |
| -        3rd tertile (10-28) | Ref | Ref | Ref |
| Greene subscale: vasomotor |  |  |  |
| -        <=2 | 0.36(0.18, 0.71) | 0.42(0.15, 1.2) | 0.38(0.16, 0.94) |
| -        3-6 | Ref | Ref | Ref |
| Greene subscale: somatic |  |  |  |
| -        1st tertile (0-2) | 0.15(0.07, 0.35) | 0.91(0.24, 3.46) | 0.54(0.16, 1.8) |
| -        2nd tertile (4-5) | 0.55(0.23, 1.29) | 1.4(0.36, 5.45) | 1.31(0.42, 4.06) |
| -        3rd tertile (6-19) | Ref | Ref | Ref |
| Greene subscale: sexual dysfunction |  |  |  |
| -        no or a little | 0.47(0.24, 0.92) | 0.4(0.14, 1.12) | 0.51(0.21, 1.23) |
| -        else | Ref | Ref | Ref |
| Pain |  |  |  |
| -        1st tertile (0-62) | 3.02(1.32, 6.88) | 1.29(0.35, 4.74) | 1.39(0.45, 4.32) |
| -        2nd tertile (63-74) | 1.76(0.63, 4.93) | 0.73(0.15, 3.67) | 1.06(0.28, 3.99) |
| -        3rd tertile (75-100) | Ref | Ref | Ref |
| **Poor total/global sleep** |  |  |  |
| Age |  |  |  |
| -        <45 year | 0.9(0.47, 1.73) | 1.19(0.35, 4.08) | 1.33(0.47, 3.74) |
| -        >= 45 years | Ref | Ref | Ref |
| Country of birth |  |  |  |
| -        Australia | 1.5(0.77, 2.94) | 1.25(0.44, 3.59) | 1.61(0.65, 3.99) |
| -        Elsewhere | Ref | Ref | Ref |
| Marital status |  |  |  |
| -        Married or de facto | 0.81(0.23, 2.87) | 1.92(0.21, 17.29) | 1.91(0.3, 12.21) |
| -        -Else | Ref | Ref | Ref |
| Employment status |  |  |  |
| -        Employed | 0.42(0.16, 1.14) | 0.35(0.07, 1.65) | 0.46(0.13, 1.69) |
| -        Else | Ref | Ref | Ref |
| Education |  |  |  |
| -        Low | 1.57(0.5, 4.96) | 1.47(0.17, 13) | 1.18(0.2, 6.96) |
| -        Intermediate | 1.25(0.64, 2.45) | 0.55(0.17, 1.76) | 0.6(0.22, 1.63) |
| -        High | Ref | Ref | Ref |
| Income |  |  |  |
| -        Less than $20,000 | 0.39(0.03, 4.41) | 1(0, 0) | 0.17(0, 10.17) |
| -        $20,000 - $80,000 | 1.45(0.7, 3.01) | 1.03(0.3, 3.48) | 0.78(0.27, 2.24) |
| -        Above $80,000 | Ref | Ref | Ref |
| Self-reported BMI |  |  |  |
| -        Obese | 1.76(0.83, 3.75) | 1.3(0.38, 4.45) | 1.88(0.63, 5.62) |
| -        Else | Ref | Ref | Ref |
| Menopausal status |  |  |  |
| -        Pre-menopausal | 1.87(0.56, 6.2) | 2.32(0.35, 15.62) | 4.46(0.92, 21.58) |
| -        Peri menopausal | 0.57(0.23, 1.41) | 0.45(0.1, 2.15) | 0.26(0.06, 1.06) |
| -        Post-menopausal | Ref | Ref | Ref |
| Greene subscale: psychological |  |  |  |
| -        1st tertile (0-5) | 0.07(0.03, 0.2) | 0.11(0.03, 0.51) | 0.06(0.02, 0.24) |
| -        2nd tertile (6-9) | 0.2(0.08, 0.55) | 0.11(0.02, 0.54) | 0.1(0.03, 0.4) |
| -        3rd tertile (10-28) | Ref | Ref | Ref |
| Greene subscale: vasomotor |  |  |  |
| -        <=2 | 0.39(0.2, 0.74) | 0.37(0.13, 1.1) | 0.49(0.2, 1.18) |
| -        3-6 | Ref | Ref | Ref |
| Greene subscale: somatic |  |  |  |
| -        1st tertile (0-2) | 0.18(0.08, 0.41) | 0.82(0.18, 3.75) | 0.99(0.28, 3.43) |
| -        2nd tertile (4-5) | 0.35(0.13, 0.93) | 0.88(0.2, 3.9) | 1.32(0.36, 4.83) |
| -        3rd tertile (6-19) | Ref | Ref | Ref |
| Greene subscale: sexual dysfunction |  |  |  |
| -        no or a little | 0.47(0.24, 0.9) | 0.68(0.23, 2.02) | 0.71(0.29, 1.75) |
| -        else | Ref | Ref | Ref |
| Pain |  |  |  |
| -        1st tertile (0-62) | 6.07(2.8, 13.17) | 10.91(2.48, 48.04) | 5.94(1.86, 18.92) |
| -        2nd tertile (63-74) | 3.54(1.4, 8.98) | 6.07(1.45, 25.41) | 3.77(1.17, 12.14) |
| -        3rd tertile (75-100) | Ref | Ref | Ref |
