# Supplementary material for: Prevalence and predictors of sleep problems in women following a cancer diagnosis: results from the women’s wellness after cancer program
Source: J Cancer Surviv. 2023 Feb 24;18(3):960–71. doi: 10.1007/s11764-023-01346-9 (PMC11082004; doi:10.1007/s11764-023-01346-9)
Supplement: Supplementary file 2 — Supplementary file2 (DOCX 19 KB) [file 11764_2023_1346_MOESM2_ESM.docx]

**Supplementary Table 2: Proportion of participants with sleep problems across different groups of participant characteristics (not included in analysis) among cancer treated women**

|  | **Sleep indicators; % (group specific N)** | | | | |
| --- | --- | --- | --- | --- | --- |
| **Covariates** | **Insufficient sleep duration; N=345** | **Poor sleep quality; N=345** | **Poor sleep efficiency; N=330** | **Frequent sleep disturbance; N=193** | **Clinically significant sleep disturbance; N=163** |
| **Physical activity** |  |  |  |  |  |
| - **Low (<600 MET.min/wk)** | 49.2 (65) | 45.5 (66) | 35.9 (64) | 27.03(37) | 66.7 (33) |
| - **Moderate (600-1199 MET.min/wk)** | 40.6 (64) | 43.9 (66) | 32.3 (62) | 22.86(35) | 58.1 (31) |
| - **High (1200+ MET.min/wk)** | 35.7 (143) | 31.0 (142) | 19.9 (136) | 24.72(89) | 53.4 (73) |
| - **Missing** | 37.0 (73) | 40.9 (71) | 33.8 (68) | 34.38(32) | 65.4 (26) |
| **Greene total score** |  |  |  |  |  |
| - **1st tertile** | 20.0 (65) | 10.8 (65) | 8.3 (60) | 8.82(34) | 22.2 (27) |
| - **2nd tertile** | 36.2 (152) | 34.2 (152) | 21.9 (146) | 22.73(88) | 58.4 (77) |
| - **3rd tertile** | 65.7 (70) | 66.2 (71) | 52.9 (68) | 46.51(43) | 88.9 (36) |
| - **Missing** | 37.9 (58) | 45.6 (57) | 35.7 (56) | 28.57(28) | 56.5 (23) |
| **CES-D score** |  |  |  |  |  |
| - **No depression** | 35.7 (216) | 30.6 (216) | 21.7 (207) | 19.5 (118) | 48.1 (104) |
| - **Yes-clinical depression** | 53.0 (83) | 61.5 (83) | 47.5 (80) | 43.1 (51) | 85.4 (41) |
| - **Missing** | 32.6 (46) | 32.6 (46) | 23.3 (43) | 25.0 (24) | 61.1 (18) |
| **Zung self-rating anxiety score** |  |  |  |  |  |
| - **Normal** | 38.1 (273) | 33.6 (271) | 26.6 (263) | 22.3 (157) | 56.8 (132) |
| - **Moderate to extreme anxiety** | 73.9 (23) | 83.3 (24) | 50.0 (22) | 50.0 (14) | 100.0 (11) |
| - **Missing** | 30.6 (49) | 42.0 (50) | 26.7 (45) | 40.9 (22) | 50.0 (20) |

Note. These variables were not included in analysis due to multicollinearity or excessive missing data (i.e., >20%). See data analysis section for more information
