# Supplementary material for: Prevalence and predictors of sleep problems in women following a cancer diagnosis: results from the women’s wellness after cancer program
Source: J Cancer Surviv. 2023 Feb 24;18(3):960–71. doi: 10.1007/s11764-023-01346-9 (PMC11082004; doi:10.1007/s11764-023-01346-9)
Supplement: Supplementary file 3 — Supplementary file3 (DOCX 28 KB) [file 11764_2023_1346_MOESM3_ESM.docx]

**Supplementary Table 3: Predictors of sleep related indicators among cancer treated women (Best predictive subset model): complete vs missing imputed analysis**

|  | **OR (95% CI)** | |
| --- | --- | --- |
| **Predictors** | **Adjusted (Complete case)** | **Adjusted (Missing imputed)** |
| **Insufficient sleep duration** |  |  |
| Education |  |  |
| - Low | 1.28(0.52, 3.18) | 1.52(0.66, 3.49) |
| - Intermediate | 2.16(1.29, 3.63) | 2.17(1.32, 3.57) |
| - High | Ref | Ref |
| Greene subscale: psychological |  |  |
| - 1st tertile (0-5) | 0.39(0.21, 0.72) | 0.39(0.21, 0.72) |
| - 2nd tertile (6-9) | 0.45(0.25, 0.82) | 0.51(0.29, 0.89) |
| - 3rd tertile (10-28) | Ref | Ref |
| Greene subscale: vasomotor |  |  |
| - <=2 | 0.47(0.29, 0.77) | 0.46(0.29, 0.75) |
| - 3-6 | Ref | Ref |
| Pain |  |  |
| - 1st tertile (0-62) | 2.02(1.12, 3.67) | 1.72(0.98, 3.04) |
| - 2nd tertile (63-74) | 2.22(1.08, 4.57) | 1.99(0.99, 4.01) |
| - 3rd tertile (75-100) | Ref | Ref |
| **Poor sleep quality** |  |  |
| Greene subscale: psychological |  |  |
| - 1st tertile (0-5) | 0.14(0.07, 0.27) | 0.14(0.07, 0.28) |
| - 2nd tertile (6-9) | 0.34(0.19, 0.61) | 0.35(0.19, 0.62) |
| - 3rd tertile (10-28) | Ref | Ref |
| Greene subscale: vasomotor |  |  |
| - <=2 | 0.59(0.35, 0.99) | 0.59(0.35, 0.97) |
| - 3-6 | Ref | Ref |
| Pain |  |  |
| - 1st tertile (0-62) | 2.79(1.48, 5.25) | 2.42(1.33, 4.42) |
| - 2nd tertile (63-74) | 1.5(0.68, 3.31) | 1.43(0.67, 3.08) |
| - 3rd tertile (75-100) | Ref | Ref |
| **Poor sleep efficiency** |  |  |
| Marital status |  |  |
| - Married or de facto | 0.77(0.41, 1.48) | 0.88(0.47, 1.64) |
| - -Else | Ref | Ref |
| Education |  |  |
| - Low | 2.26(0.85, 6.01) | 2.41(0.98, 5.93) |
| - Intermediate | 1.98(1.1, 3.55) | 1.86(1.06, 3.25) |
| - High | Ref | Ref |
| Greene subscale: psychological |  |  |
| - 1st tertile (0-5) | 0.14(0.06, 0.3) | 0.15(0.07, 0.32) |
| - 2nd tertile (6-9) | 0.41(0.22, 0.76) | 0.48(0.26, 0.86) |
| - 3rd tertile (10-28) | Ref | Ref |
| Greene subscale: vasomotor |  |  |
| - <=2 | 0.52(0.29, 0.91) | 0.53(0.31, 0.92) |
| - 3-6 | Ref | Ref |
| **Sleep disturbance** |  |  |
| Age |  |  |
| - <45 year | 0.18(0.07, 0.5) | 0.25(0.1, 0.62) |
| - >= 45 years | Ref | Ref |
| Marital status |  |  |
| - Married or de facto | 0.18(0.04, 0.85) | 0.23(0.06, 0.91) |
| - -Else | Ref | Ref |
| Greene subscale: psychological |  |  |
| - 1st tertile (0-5) | 0.28(0.1, 0.75) | 0.27(0.1, 0.7) |
| - 2nd tertile (6-9) | 0.24(0.09, 0.64) | 0.3(0.12, 0.72) |
| - 3rd tertile (10-28) | Ref | Ref |
| Greene subscale: vasomotor |  |  |
| - <=2 | 0.34(0.14, 0.79) | 0.4(0.18, 0.88) |
| - 3-6 | Ref | Ref |
| Greene subscale: sexual dysfunction |  |  |
| - no or a little | 0.41(0.18, 0.95) | 0.52(0.24, 1.13) |
| - else | Ref | Ref |
| **Poor total/global sleep** |  |  |
| Employment status |  |  |
| - Employed | 0.36(0.11, 1.18) | 0.39(0.12, 1.25) |
| - Else | Ref | Ref |
| Greene subscale: psychological |  |  |
| - 1st tertile (0-5) | 0.1(0.03, 0.32) | 0.11(0.04, 0.3) |
| - 2nd tertile (6-9) | 0.1(0.03, 0.35) | 0.18(0.06, 0.54) |
| - 3rd tertile (10-28) | Ref | Ref |
| Greene subscale: vasomotor |  |  |
| - <=2 | 0.46(0.19, 1.09) | 0.52(0.23, 1.17) |
| - 3-6 | Ref | Ref |
| Pain |  |  |
| - 1st tertile (0-62) | 8.1(2.87, 22.89) | 4.87(1.98, 11.97) |
| - 2nd tertile (63-74) | 4.3(1.33, 13.95) | 3.38(1.17, 9.73) |
| - 3rd tertile (75-100) | Ref | Ref |
